# “Dead Cells Talking”: The Silent Form of Cell Death Is Not so Quiet

**DOI:** 10.1155/2012/453838

**Published:** 2012-08-07

**Authors:** Richard Jäger, Howard O. Fearnhead

**Affiliations:** ^1^Department of Natural Sciences, Bonn-Rhein-Sieg University of Applied Sciences, 53359 Rheinbach, Germany; ^2^Department of Pharmacology and Therapeutics, NUI Galway, Galway, Ireland

## Abstract

After more than twenty years of research, the molecular events of apoptotic cell death can be succinctly stated; different pathways, activated by diverse signals, increase the activity of proteases called caspases that rapidly and irreversibly dismantle condemned cell by cleaving specific substrates. In this time the ideas that apoptosis protects us from tumourigenesis and that cancer chemotherapy works by inducing apoptosis also emerged. Currently, apoptosis research is shifting away from the intracellular events within the dying cell to focus on the effect of apoptotic cells on surrounding tissues. This is producing counterintuitive data showing that our understanding of the role of apoptosis in tumourigenesis and cancer therapy is too simple, with some interesting and provocative implications. Here, we will consider evidence supporting the idea that dying cells signal their presence to the surrounding tissue and, in doing so, elicit repair and regeneration that compensates for any loss of function caused by cell death. We will discuss evidence suggesting that cancer cell proliferation may be driven by inappropriate or corrupted tissue-repair programmes that are initiated by signals from apoptotic cells and show how this may dramatically modify how we view the role of apoptosis in both tumourigenesis and cancer therapy.

## 1. Introduction

The idea that apoptosis is a homeostatic mechanism that can act as a counterbalance to cell proliferation is central to our understanding of programmed cell death (reviewed by Melino et al. [1]). Inherent in this idea is the existence of cell-cell signalling that communicates a cell's behaviour and fate to those surrounding it. We now have a detailed understanding of how a range of different stimuli can induce apoptosis in a cell, which includes the key molecules within a dying cell that either transduce death signals or actively destroy the condemned cell. The common theme that emerges is the activation of specific intracellular proteases (the caspases) which cleave critical substrates and thus generate the typical morphological and biochemical changes of apoptosis. Apoptotic stimuli first trigger the assembly of protein complexes that are activation platforms for initiator caspases (such as caspase-8 and -9). Activated initiators then cleave and activate the precursors of the executioner caspases (such as caspase-3 and -7) that subsequently act on the various cellular substrates [2]. This valuable knowledge provides a robust mechanistic understanding of the cell-intrinsic mechanisms of death, but it does not explain the cell-cell communication that couples proliferation and cell death. 

Apoptosis has traditionally been called the silent cell death because it does not trigger an inflammatory response, but more recent studies have uncovered evidence of paracrine signals originating from apoptotic cells. These studies, which are from several different model systems, suggest that the appearance of apoptotic cells can constitute a signal for the proliferation of stem or progenitor cell populations and that this compensatory proliferation is vital for the repair and regeneration of damaged tissue. Thus, apoptosis is far from being a silent cell death, and, even in death, apoptotic cells seem to play a key function in tissue homeostasis.

## 2. Apoptosis, Caspases, and Repair and Regeneration

**Figure 1:**
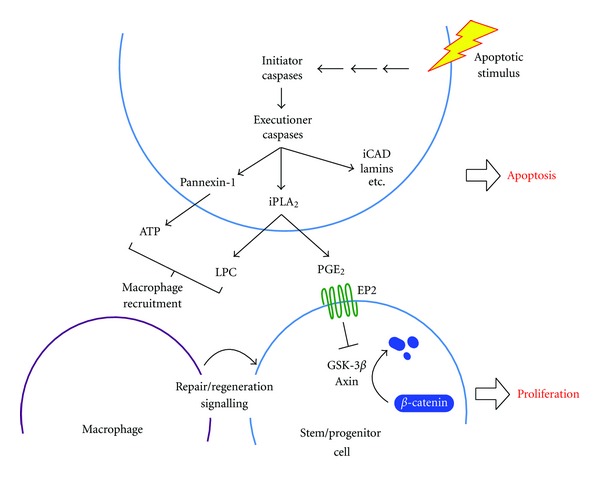
Apoptotic stimuli activate caspases, triggering the proteolysis of a large number of intracellular substrates. The cleavage of many of these, including iCAD and lamins, is necessary for the morphological and biochemical changes of apoptosis. Other substrates have as yet undefined roles, while the cleavage of iPLA_2_ is critical for the paracrine signalling that induces compensatory proliferation. Cleavage of iPLA_2_ increases its activity, so raising the levels of PGE_2_ and LPC. PGE_2_ in turn activates EP2 G protein-coupled receptors on stem or progenitor cells. Intracellular signalling downstream of EP2 activates *β*-catenin and leads to cell proliferation. LPC and ATP may indirectly induce compensatory proliferation through the recruitment of macrophages.

Studies in *Drosophila*, *Xenopus*, planaria, and *Hydra* have revealed a role for apoptotic cells in repair and regeneration [3–6], and the first clear evidence of its role in mammalian repair and regeneration came from a study using caspase-null mice [7]. Skin and liver regeneration was investigated by studying the rate of wound healing in the skin and the rate of liver regeneration following partial hepatectomy. Li et al. reported that the loss of caspase-3 and/or caspase-7 markedly reduced the rate of tissue repair in both instances. Follow-up* in vitro* experiments showed that apoptotic cells released prostaglandin E2 (PGE_2_) in a caspase-dependent fashion and that this induced the proliferation of various stem cell types [7] (Figure 1).

A potential role for apoptosis in the repair and regeneration of pancreatic *β*-cells has also been uncovered [8]. In this model, caspase activity was also required, although the signal from apoptotic cells appeared to be membrane-bound microparticles derived from blebbing cells, and the specific signalling molecule or molecules involved have yet to be identified. Nonetheless, these data show that there are likely to be several different types of signals driving regeneration that originate from apoptotic cells.

## 3. Prostaglandins: The Role of Caspases in Their Generation and Their Effects on Target Cells

Prostaglandins have already been established as playing a role in tissue repair [9, 10], although their increase in concentration was not linked to apoptotic cells. Prostaglandins are eicosanoids, a group of rapidly synthesized signalling molecules that play critical roles in inflammation and other processes. The rate-limiting step in eicosanoid generation is the conversion of membrane phospholipids to arachidonic acid by phospholipase A_2_. Prostaglandins are subsequently formed by the action of cyclooxygenase enzymes on arachidonic acid and can then elicit a range of effects by binding to G protein-coupled receptors on target cells. They are also rapidly inactivated, and so, without persistent stimulus for their generation, their signalling is short-lived. During apoptosis arachidonic acid is released due to the caspase-3-dependent cleavage of Ca^2+^-independent phospholipase A_2_ (iPLA_2_), an event that increases the catalytic activity of iPLA_2_ and raises PGE_2_ levels [11].

## 4. PGE_2_ and Activation of ***β***-Catenin

It is not yet clear how PGE_2_ induces compensatory proliferation, but there are clues that implicate *β*-catenin signalling. In both zebrafish and mice, stem cell regulation by Wnt/*β*-catenin signalling has been shown to require PGE_2_; thus *β*-catenin activation by PGE_2_ has been described as a master regulator of stem cells in tissue regeneration [12]. 

Of the four distinct G protein-coupled receptors to which PGE_2_ binds (EP1-EP4; [13]), EP2 has been shown to activate *β*-catenin. In the absence of activating stimuli, *β*-catenin is found in a cytoplasmic complex with the proteins axin, adenomatous polyposis coli (APC), and glycogen synthase kinase-3*β* (GSK-3*β*). In this complex GSK-3*β* phosphorylates *β*-catenin, inducing its ubiquitination and rapid proteosomal degradation, so preventing *β*-catenin signalling [14]. PGE_2_ binding to EP2 leads to dissociation of the G protein subunits G_*α*s_ and *βγ*. While G_*α*s_ binds to axin, leading to the release of GSK-3*β*, the *βγ* subunits activate AKT which inactivates GSK-3*β* by phosphorylation [15].

To this point the focus has been on the role of apoptosis in normal tissue repair and regeneration; however, there are many obvious links to tumourigenesis. For example, partial hepatectomy promotes tumour formation, APC is a tumour suppressor, and PGE_2_ is known to contribute to tumour promotion. *In vitro* PGE_2_ increases cellular proliferation and induces other cell behaviour typical of cancer cells such as reduced expression of E-cadherin, reduced apoptosis, and anchorage-independent growth [16]. In addition, EP2-null mice form fewer lung and skin tumours than wild-type mice when exposed to chemical carcinogens [17, 18]. Similarly, mice lacking microsomal prostaglandin E synthase-1 show reduced colon tumour formation [19]. Since spontaneous apoptosis of tumour cells is frequently occurring within tumours [20], it is possible that these phenotypes are, at least in part, due to an impaired compensatory proliferation of tumor cells. Therefore, these observations prompt the following question: what is the role of apoptosis-induced compensatory proliferation in cancer development and cancer treatment?

## 5. Puma, Apoptosis, and Tumourigenesis

The idea that perturbing the balance between death and proliferation causes disease emerged very early within the apoptosis research field [21]. Perhaps the best example is cancer; the observation that cancers often acquire mutations that prevent apoptosis has been explained by the survival of precancerous cells (that would otherwise die) giving rise to neoplasia. However, new data, building on the ideas of apoptosis-induced compensatory proliferation in normal repair and regeneration, show that the role of apoptosis in tumourigenesis does not end with the death of cell.

The first pieces of evidence come from two studies of lymphoma formation performed in PUMA-null mice [22, 23]. In both studies lymphomagenesis was induced by *γ*-irradiation. Both groups also showed that *γ*-irradiation-induced apoptosis was markedly reduced in PUMA-null animals. This is rather unsurprising as PUMA is a proapoptotic (BH3-only) member of the Bcl-2 family and a key player in the mitochondrial apoptotic pathway activated by DNA damage [24]. PUMA acts by either inhibiting Bcl-2 function or by activating Bax and Bak, so triggering the release of cytochrome *c* from mitochondria. The subsequent Apaf-1-dependent caspase activation induces apoptosis [25].

What is surprising is that, following *γ*-irradiation, PUMA-null mice showed decreased tumour incidence. Interestingly, irradiation of wild-type mice resulted in compensatory proliferation of a population of hematopoietic stem/progenitor cells, but this proliferation was decreased in PUMA-null animals. The idea that PUMA-dependent apoptotic cell death was driving the compensatory proliferation is driven home by one further experiment: Michalak et al. induced leukocyte apoptosis in PUMA-null animals with a glucocorticoid (which causes PUMA-independent apoptosis) and by this means restored *γ*-irradiation-induced lymphomagenesis in these animals [22].

Another key piece of evidence comes from a mouse model of chemical carcinogenesis. Diethylnitrosamine (DEN) is an DNA-alkylating agent that induces apoptosis and is also a known hepatocarcinogen. Qiu et al. [26] showed that DEN treatment of wild-type mice induced hepatocyte apoptosis which was reduced in PUMA-deficient mice.

Just as was seen in *γ*-irradiation-induced lymphomagenesis, PUMA-null mice showed both decreased tumour incidence and also decreased tumour size. Qiu et al. also observed that DEN induced a PUMA-dependent compensatory proliferation in the liver. The authors concluded that the DEN-induced apoptosis causes increased, or compensatory, proliferation that drives hepatocarcinogenesis, an idea consistent with the observations that apoptotic hepatocytes are often surrounded by proliferating cells following DENtreatment [27]. Indeed, the role of compensatory proliferation in DEN-induced hepatocellular carcinoma is well supported by other studies, although these studies implicate a major role for inflammation rather than apoptosis in compensatory proliferation [27, 28]. Interestingly, all these studies are foreshadowed by observations linking carbon tetrachloride-induced hepatotoxicity and compensatory proliferation to carcinogenesis, although again, the causal role of apoptotic cells was not investigated [29].

The signalling events involved in inducing compensatory proliferation in lymphomagenesis and hepatocarcinogenesis remain unexplored, so there is as yet no direct link to PGE_2_, the key signalling molecule identified by Li et al. [7]. Nonetheless, all the data are consistent with a model in which carcinogen-induced DNA damage kills some cells by apoptosis and that these apoptotic cells generate a proproliferation signal. This signal then promotes tumourigenesis by acting on surrounding cells that have acquired oncogenic mutations as a result of sublethal levels of DNA damage.

## 6. Radiotherapy, Apoptosis, and Tumour Repopulation

A similarly provocative idea has been put forward by the same group that originally described the role of mammalian caspases in regeneration [30]. Following radiotherapy to kill cancer cells, there is a rapid proliferation of the surviving cancer cells that repopulate the tumour. Huang et al. [30] investigated the mechanism of this repopulation using a mouse model and found that it was caspase-3-dependent and, like tissue regeneration, involved PGE_2_. Interestingly, the overexpression of caspase-3 in MCF-7 cells (which lack caspase-3) increased tumour growth in a xenograft model, indicating that, even in the absence of therapy, caspase-3 plays an unexpected protumourigenic role. The mechanism underlying the increased rate of tumour growth seen with unirradiated MCF-7 cells overexpressing caspase-3 was not investigated. Huang et al. speculated that the caspase activation accompanying spontaneous apoptotic cell death may be the cause of this increased rate in tumour growth. 

In the same study the role of stromal cells in caspase-3-dependent tumour repopulation was assessed using a syngenic tumour cell line transplanted into wild-type and caspase-3-null mice. Cells transplanted into wild-type mice formed larger tumours compared to the cells transplanted into null animals, suggesting that the tumour cell-stromal interactions supporting tumour growth are also caspase-3 dependent.

Lastly, Huang et al. [30] investigated the relationship between levels of activated caspase-3 in clinical tumour samples and patient outcome. This analysis revealed a positive correlation between expression of active caspase-3 in tumours and poorer prognosis in patients with head and neck carcinoma and advanced breast cancer, adding weight to the counterintuitive idea that increased apoptosis contributes to disease progression. 

**Figure 2:**
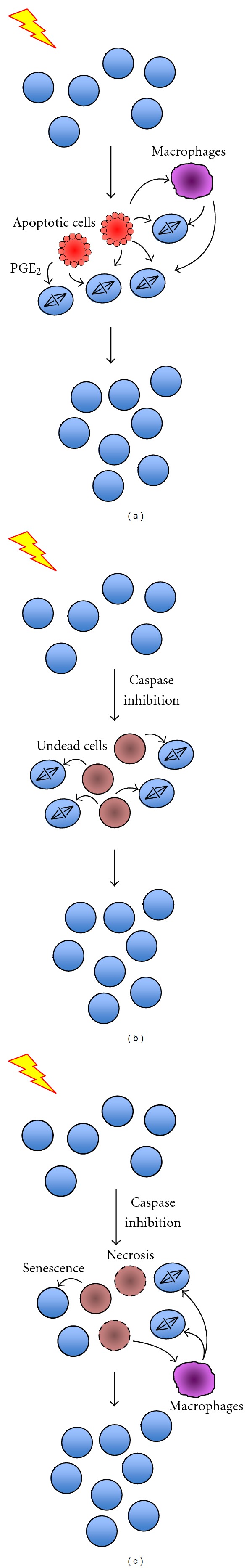
Radio- or chemotherapy induces cancer cell apoptosis generating proliferation signals that drive the rapid proliferation of surviving cancer cells which repopulate the tumour by generating signals that act directly on stem/progenitor cells or by recruiting macrophages (a). The role of PUMA in compensatory proliferation has led to the suggestion that blocking caspase-mediated cleavage of iPLA_2_ with small molecule caspase inhibitors may improve patient outcomes by preventing this compensatory proliferation. However the general applicability of this model is uncertain for several reasons. Firstly, in *Drosophila* development blocking apoptosis in some circumstances produces undead cells whose persistent signalling increases compensatory proliferation (b). Secondly, while caspase inhibitors block apoptosis, the irradiated or drug-treated cells may still die by nonapoptotic processes or become senescent, which may induce compensatory proliferation as well (c).

Again, the idea of compensatory proliferation induced by apoptotic cells was evoked to explain these observations. In such a model, radiotherapy and chemotherapy induces apoptosis by activating caspases, either through the “intrinsic” or mitochondrial pathway [31] or by RIP-kinase-dependent activation of caspase-8 [32]. While this apoptosis does indeed kill the cancer cells, the unintended consequence is the inevitable increase in proproliferation signalling from the dying cells. Unfortunately, the result is not normal tissue regeneration, but the rapid proliferation of surviving cancer cells that repopulate the tumour (Figure 2(a)). Thus, the very mechanism of cell death induced by most currently employed cancer chemotherapy can limit the efficacy of that therapy.

This provocative idea led Huang et al. to the counterintuitive suggestion that treatment of patients with radio- or chemotherapy supplemented by caspase inhibitors may improve patient outcomes by blocking caspase-mediated cleavage of iPLA_2_ and so prevent compensatory proliferation. While the toxicity of the caspase inhibitor zVAD-fmk precludes its *in vivo* use [33], inhibitors such as Q-VD-OPh are available and suitable for this purpose [34]. These caspase inhibitors will, of course, also block cancer cell apoptosis, but there is reason to think that the combination therapy would still improve anticancer activity.

## 7. Caspase Activity Determines the Mode of Cell Death, but Blocking Activity Does Not Necessarily Keep Cells Alive

Caspase activity induces apoptosis but blocking caspase activity does not necessarily rescue cells from cell death. While caspase inhibitors prevent the appearance of the morphological and biochemical features of apoptosis induced by anticancer agents, affected cells still die by necrotic or necroptotic processes. Thus caspase activity can determine the mode of cell death but not necessarily the choice between death and survival [35].

Anticancer therapy can also induce necrotic cell death directly [36] or induce senescence [37]. Necrotic cell death can promote tumourigenesis through induction of inflammation [38], and senescence is known to generate paracrine signals that can promote tumourigenesis such as IL-6 and -8 [39, 40] (Figures 2(b) and 2(c)). However, the data of Huang et al. [30] from caspase-null animals suggest that signals derived from apoptotic cells following radiotherapy are the most important. This is either because apoptosis was the predominant cell fate induced by radiotherapy or because paracrine signals from apoptotic cells are more potent tumour promoters. Whether this is true of other cancer therapies still needs to be tested through further investigation of apoptotic and nonapoptotic cell death and of the generation of tumour-promoting paracrine signalling molecules in tumours. Testing the generality of these conclusions is particularly important as studies of compensatory proliferation in Drosophila development show that blocking apoptosis in some contexts can result in “undead” cells whose persistent signaling increases, rather than decreases, proliferation [41–45]. Whatever the outcome of such studies, a consideration of cell death-derived signalling appears to be necessary when weighing the factors that limit the efficacy of chemotherapy.

Targeting caspase activity may be attractive as these proteases drive the apoptotic process and so the production of signals that induce compensatory proliferation. However, interfering with the signals themselves may also work, and this is an attractive alternative as there are already a range of nonsteroidal anti-inflammatory drugs (NSAIDs) that affect prostaglandin production. Indeed, NSAIDs have been, and continue to be, tested for anticancer therapy although not for the reasons discussed here (there are several different rationales for testing the anticancer activity of NSAIDs from their antiangiogenic activity to their ability to reduce chemotherapy-induced neuropathy [46, 47]). The limitation of this approach is that other potent regeneration/repair signals emanating from apoptotic cells, such as microparticles, may not be sensitive to NSAIDs, and targeting the apoptotic process itself might therefore be more efficacious.

## 8. A Conspiracy between Apoptotic Cells and Immune Cells May Promote Tumor Development

So far we have discussed how apoptotic cells talk to stem or progenitor cell populations and so induce tissue repair. However, there is good evidence that other cell types are listening to apoptotic cells, notably phagocytes. Thus, apoptotic cells initiate their own clearance by actively releasing several soluble factors that attract phagocytic cells. These so-called find-me signals include the nucleotides ATP and UTP [48, 49], lysophosphatidylcholine (LPC) [50], and sphingosine-1-phosphate (S1P) [51](reviewed in [52]). Interestingly, the release of two of these factors has been shown to depend on caspase activity within apoptotic cells. Release of ATP occurs via the membrane channel protein Pannexin-1, which becomes activated by caspase-3/7-mediated cleavage [49]. In addition caspase-mediated activation of iPLA_2_ is not only involved in raising PGE_2_ levels (as discussed above) but at the same time leads to LPC production by hydrolysis of membrane phosphatidylcholine [50]. ATP and LPC are linked primarily with attracting professional phagocytes such as macrophages to facilitate clearance of apoptotic bodies. However, these macrophages also represent a likely source of wound healing signals [50], such as platelet-derived growth factors and TGF-*β*1, that stimulate proliferation of surrounding epithelial cells and fibroblasts and may also act on tumor cells themselves [53, 54]. In fact, there is accumulating evidence from a number of tumor models to suggest that these macrophages contribute to tumor cell proliferation and metastasis [55, 56] and thus enhance the progression of tumors towards malignancy. So, apoptotic cells appear to promote tissue regeneration and repair processes by at least two mechanisms: one is a mechanism involving the generation of signals that act directly on stem and/or progenitor cells, and the other is an indirect mechanism that involves recruitment of macrophages which subsequently release proproliferative factors.

## 9. How Do These New Ideas Fit with the Predominant View of Apoptosis in Cancer?

The activation of many oncogenes can induce apoptosis, and the loss of tumour suppressors can prevent apoptosis, and this leads to the predominant view that apoptosis protects us from cancer by removing precancerous cells and that cancer can only occur when the apoptotic process is compromised [48]. Similarly, there is much evidence to show that radiotherapy and chemotherapeutic drugs induce apoptosis [31], although this is not the only fate that can be induced.

At first glance, the new idea that apoptosis promotes tumourigenesis and limits the efficacy of therapy challenges the predominant viewpoint, but the apparently conflicting roles of apoptosis are not at all incompatible. Firstly, a maelstrom of often conflicting changes occurs during tumourigenesis but the emergence of a tumour represents selection of cells which have struck a balance between these changes, that is compatible with cell proliferation. Depending on the sequence of events that lead to a tumour being formed, it is possible that at early stages apoptotic cell death is a promoting event but that the acquisition of mutations that compromise apoptosis at a later stage drives disease progression. Indeed, apoptosis or inflammation may increase PGE_2_ levels at early stages of the disease, but autocrine PGE_2_ production can occur at a later stage of the disease [49]. Similarly, effective cancer treatment is defined by the extent of tumour regression, and identification of an effective therapy suggests that balance between apoptotic cell death and proproliferation signals from apoptotic cells favours net cell loss. The new findings suggest that interfering with apoptosis may push this balance even further towards cell loss by blocking compensatory proliferation and so enhance the efficacy of therapy.

## 10. Final Thoughts

The studies outlined here show that apoptotic cells, far from being silent, signal their presence to stem and progenitor cells in surrounding tissue and in doing so elicit repair and regeneration. This coupling of cell loss with a compensatory proliferation and differentiation is consistent with current models of the role of apoptosis in tissue homeostasis. The obvious corollary is that the activation of corrupted proliferation and tissue repair programmes in stem cells can contribute to tumourigenesis, and this is also being borne out by experimental data.

Intriguing and important as the new findings are, we should end with some words of caution. The general relevance of the new roles of apoptosis in tumourigenesis and therapy has not been tested. Given the diversity of cancers, it seems likely that the development of some tumours may involve much apoptosis-driven tumour promotion, while in others it may be of little importance.

Similarly, it has been assumed that compensatory proliferation induced by radiotherapy will also be induced by cytotoxic therapy that triggers apoptosis and that the new findings are therefore relevant to other cancer therapies [30]. This has yet to be established, and even if it is, considering the wide range of different chemistries, different mechanisms of action and different pharmacokinetics and pharmacodynamics associated with chemotherapy, some drugs may be better or poorer inducers of compensatory proliferation.

One obvious difference between radiotherapy and chemotherapy is that radiotherapy is typically delivered for a very short period of time compared to chemotherapy. Thus, after radiotherapy, compensatory proliferation occurs in the absence of a continuing apoptotic stimulus. In contrast, a chemotherapeutic drug may persist at pharmacologically relevant concentrations over the period of time that encompasses compensatory proliferation. In this instance, a cytotoxic drug may kill the activated stem cells as they proliferate, leading to a second round of cell death. These deaths in turn may activate a further round of compensatory proliferation and so on, until the drug's concentration falls to subtoxic levels. The outcome in this situation is hard to predict but will be affected by the sensitivity of the stem cells to the drug, the proportion of cells that are able to respond to the paracrine signals and the pharmacokinetics of the particular drug.

Finally, there are also indications that PGE_2_ is not the only method of “beyond-the-grave” communication undertaken by apoptotic cells [8], nor is *β*-catenin signalling the only pathway implicated in compensatory proliferation [3, 26]. Finding out just how noisy the supposedly “silent cell death” really is will help in understanding how signals from apoptotic cells might regulate normal and pathological processes and build a more complete picture of the apoptotic process.
